# Supplementary material for: The noisy voter model on complex networks
Source: Sci Rep. 2016 Apr 20;6:24775. doi: 10.1038/srep24775 (PMC4837380; doi:10.1038/srep24775)
Supplement: Supplementary Information [file srep24775-s1.pdf]

Supplementary Information for:

The noisy voter model on complex networks

Adrián Carro[1,*], Raúl Toral[1], and Maxi San Miguel[1]

[1]IFISC (CSIC-UIB), Instituto de Física Interdisciplinar y Sistemas Complejos, Campus Universitat de les Illes Balears, E-07122, Palma de Mallorca, Spain
[*]adrian.carro@ifisc.uib-csic.es

Contents

1 Master equation 1
2 Equation for the time evolution of the first-order moments $\langle s_i \rangle$ 2
3 Equation for the time evolution of the second-order cross-moments $\langle s_i s_j \rangle$ 4
4 Variance of n 5
5 Asymptotic approximations for the variance of n 8
6 Critical point approximation 10
7 Order parameter: the interface density ρ 10
8 Autocorrelation function of n 11
9 Suppementary Figure S1 13
References 13

1 Master equation

We derive here a general master equation for the N-node probability distribution $P(s_1,\dots,s_N)$, where the individual node variables are binary and take the values $s_i = \{0,1\}$. Recalling that r_i^+ is the rate at which node i changes its state from $s_i = 0$ to $s_i = 1$ and r_i^- the rate at which it does so in the opposite direction, we can directly write differential equations for the probability of node i to be in state $s_i = 0$ and for its probability to be in state $s_i = 1$, respectively,

$$\begin{aligned}\frac{dP(s_i=0)}{dt} &= -r_i^+ P(s_i=0) + r_i^- P(s_i=1)\,,\\ \frac{dP(s_i=1)}{dt} &= -r_i^- P(s_i=1) + r_i^+ P(s_i=0)\,.\end{aligned} \tag{S1}$$

Introducing here the individual-node step operators E_i^{+1} and E_i^{-1}, whose effect over an arbitrary function of the state of node i, $f(s_i)$, is defined as

$$\begin{aligned}E_i^{+1}\left[f(s_i=0)\right] &= f(s_i=1)\,,\\ E_i^{+1}\left[f(s_i=1)\right] &= 0\,,\\ E_i^{-1}\left[f(s_i=0)\right] &= 0\,,\\ E_i^{-1}\left[f(s_i=1)\right] &= f(s_i=0)\,,\end{aligned} \tag{S2}$$

we can rewrite equations (S1) as

$$\begin{aligned}\frac{dP(s_i=0)}{dt} &= -r_i^+ P(s_i=0) + r_i^- E_i^{+1} P(s_i=0)\,,\\ \frac{dP(s_i=1)}{dt} &= -r_i^- P(s_i=1) + r_i^+ E_i^{-1} P(s_i=1)\,.\end{aligned} \tag{S3}$$

Multiplying these two equations, respectively, by $(1-s_i)$ and s_i, we can gather them in a single differential equation,

$$\frac{dP(s_i)}{dt} = (1-s_i)\left[-r_i^+ P(s_i) + r_i^- E_i^{+1} P(s_i)\right] + s_i\left[-r_i^- P(s_i) + r_i^+ E_i^{-1} P(s_i)\right] , \tag{S4}$$

and noticing that $(1-s_i) = E_i^{+1}[s_i]$ and $s_i = E_i^{+1}[(1-s_i)]$, we can rearrange terms as

$$\frac{dP(s_i)}{dt} = \left(E_i^{+1} - 1\right)\left[s_i r_i^- P(s_i)\right] + \left(E_i^{-1} - 1\right)\left[(1-s_i) r_i^+ P(s_i)\right] . \tag{S5}$$

Finally, we find the master equation for the N-node probability distribution $P(s_1,\ldots,s_N)$ by simply adding up the contribution of every single node $i \in [1,N]$,

$$\frac{dP(s_1,\ldots,s_N)}{dt} = \sum_{i=1}^{N}\left(E_i^{+1} - 1\right)\left[s_i r_i^- P(s_1,\ldots,s_N)\right] + \sum_{i=1}^{N}\left(E_i^{-1} - 1\right)\left[(1-s_i) r_i^+ P(s_1,\ldots,s_N)\right] . \tag{S6}$$

2 Equation for the time evolution of the first-order moments $\langle s_i \rangle$

We show, in this section, how to obtain a general equation for the time evolution of the first-order moments $\langle s_i \rangle$ [equation (4) in the main text]. Let us start by using the definition of the step operators in equation (S2) and the binary character of each individual node state variable, $s_i = \{0,1\}$, to derive, for a given function of the state of node i, $f(s_i)$, four relations which will ease later calculations. While the function f might also depend on the other variables, $f = f(s_1,...,s_i,...,s_N)$, we restrict our attention, without loss of generality, to the case $f(s_i)$. For the first two relations, we have that

$$\sum_{s_i}\left(E_i^{+1} - 1\right)\left[s_i f(s_i)\right] = \sum_{s_i}\left(E_i^{+1}\left[s_i f(s_i)\right] - s_i f(s_i)\right) = 1\cdot f(1) - 0\cdot f(0) + 0 - 1\cdot f(1) = 0 , \tag{S7}$$

and

$$\sum_{s_i}\left(E_i^{-1} - 1\right)\left[(1-s_i) f(s_i)\right] = \sum_{s_i}\left(E_i^{+1}\left[(1-s_i) f(s_i)\right] - (1-s_i) f(s_i)\right) = 0 - 1\cdot f(0) + 1\cdot f(0) - 0\cdot f(1) = 0 , \tag{S8}$$

where the sums are over the two possible values of s_i. Looking at the master equation (S6), one can understand that these two relations translate the fact that any increase in the probability of a given node being in a given state must be accompanied by a corresponding decrease in the probability of the complementary state. Regarding the other two relations, we can write

$$\begin{aligned} \sum_{s_i} s_i\left(E_i^{+1} - 1\right)\left[s_i f(s_i)\right] &= \sum_{s_i} s_i\left(E_i^{+1}\left[s_i f(s_i)\right] - s_i f(s_i)\right) \\ &= 0\cdot(1\cdot f(1) - 0\cdot f(0)) + 1\cdot(0 - 1\cdot f(1)) = -1\cdot f(1) \\ &= -\sum_{s_i} s_i f(s_i) , \end{aligned} \tag{S9}$$

and

$$\begin{aligned} \sum_{s_i} s_i\left(E_i^{-1} - 1\right)\left[(1-s_i) f(s_i)\right] &= \sum_{s_i} s_i\left(E_i^{+1}\left[(1-s_i) f(s_i)\right] - (1-s_i) f(s_i)\right) \\ &= 0\cdot(0 - 1\cdot f(0)) + 1\cdot(1\cdot f(0) - 0\cdot f(1)) = 1\cdot f(0) \\ &= \sum_{s_i}(1-s_i) f(s_i) . \end{aligned} \tag{S10}$$

Let us also introduce, for clarity, the notation $\sum_{\{s\}}$ to refer to the sum over all the possible combinations of states of all the individual nodes' variables,

$$\sum_{\{s\}} \equiv \sum_{s_1}\sum_{s_2}\cdots\sum_{s_N} , \tag{S11}$$

and $\sum_{\{s\}_j}$ to indicate the sum over all the possible combinations of states of all the variables except s_j,

$$\sum_{\{s\}_j} \equiv \sum_{s_1}\cdots\sum_{s_{j-1}}\sum_{s_{j+1}}\cdots\sum_{s_N} . \tag{S12}$$

Note that these two definitions are related by

$$\sum_{\{s\}} = \sum_{\{s\}_j} \sum_{s_j}, \tag{S13}$$

which allows us to split the sum over all possible configurations of the system into a sum over the values of one of the variables and a sum over the configurations of the rest of the system. By using the notation in (S11), the average of a given function of the states of the nodes, $f(s_1,\ldots,s_N)$, can be written as

$$\Big\langle f(s_1,\ldots,s_N)\Big\rangle = \sum_{\{s\}} f(s_1,\ldots,s_N)P(s_1,\ldots,s_N)\,. \tag{S14}$$

Using this expression and the master equation in (S6) we derive an equation for the time evolution of the average value of the state of node i,

$$\begin{aligned}\frac{d\langle s_i\rangle}{dt} &= \sum_{\{s\}} s_i \frac{dP(s_1,\ldots,s_N)}{dt}\\ &= \sum_{\{s\}}\sum_{j=1}^{N} s_i \left(E_j^{+1}-1\right)\left[s_j r_j^- P(s_1,\ldots,s_N)\right] + \sum_{\{s\}}\sum_{j=1}^{N} s_i \left(E_j^{-1}-1\right)\left[(1-s_j) r_j^+ P(s_1,\ldots,s_N)\right]\,.\end{aligned} \tag{S15}$$

Separating the terms with $j=i$ and those with $j\neq i$, we find

$$\begin{aligned}\frac{d\langle s_i\rangle}{dt} &= \sum_{\{s\}} s_i \left(E_i^{+1}-1\right)\left[s_i r_i^- P(s_1,\ldots,s_N)\right] + \sum_{\{s\}} s_i \left(E_i^{-1}-1\right)\left[(1-s_i) r_i^+ P(s_1,\ldots,s_N)\right]\\ &+ \sum_{\{s\}}\sum_{j\neq i}^{N} s_i \left(E_j^{+1}-1\right)\left[s_j r_j^- P(s_1,\ldots,s_N)\right] + \sum_{\{s\}}\sum_{j\neq i}^{N} s_i \left(E_j^{-1}-1\right)\left[(1-s_j) r_j^+ P(s_1,\ldots,s_N)\right]\,.\end{aligned} \tag{S16}$$

If we now use the relation (S13) to extract, from the general sum over $\{s\}$, the sum over the values of s_i for the terms with $j=i$, while we extract the sum over the values of s_j for the terms with $j\neq i$, we obtain

$$\begin{aligned}\frac{d\langle s_i\rangle}{dt} &= \sum_{\{s\}_i}\left[\left(\sum_{s_i} s_i \left(E_i^{+1}-1\right)\left[s_i r_i^- P(s_1,\ldots,s_N)\right]\right) + \left(\sum_{s_i} s_i \left(E_i^{-1}-1\right)\left[(1-s_i) r_i^+ P(s_1,\ldots,s_N)\right]\right)\right]\\ &+ \sum_{j\neq i}^{N}\sum_{\{s\}_j} s_i \left[\left(\sum_{s_j}\left(E_j^{+1}-1\right)\left[s_j r_j^- P(s_1,\ldots,s_N)\right]\right) + \left(\sum_{s_j}\left(E_j^{-1}-1\right)\left[(1-s_j) r_j^+ P(s_1,\ldots,s_N)\right]\right)\right],\end{aligned} \tag{S17}$$

where we can easily identify relations (S7) and (S8) for the terms with $j\neq i$, and relations (S9) and (S10) for the terms with $j=i$. In this way, we can write

$$\frac{d\langle s_i\rangle}{dt} = \sum_{\{s\}_i}\left(-\sum_{s_i} s_i r_i^- P(s_1,\ldots,s_N)\right) + \sum_{\{s\}_i}\left(\sum_{s_i}(1-s_i) r_i^+ P(s_1,\ldots,s_N)\right), \tag{S18}$$

which, after combining the sums together again, becomes

$$\frac{d\langle s_i\rangle}{dt} = \sum_{\{s\}}\left[r_i^+ - (r_i^+ + r_i^-)s_i\right] P(s_1,\ldots,s_N)\,, \tag{S19}$$

and we finally find the equation for the time evolution of the first-order moments presented in the main text,

$$\frac{d\langle s_i\rangle}{dt} = \langle r_i^+\rangle - \langle (r_i^+ + r_i^-)s_i\rangle. \tag{S20}$$

3 Equation for the time evolution of the second-order cross-moments $\langle s_i s_j \rangle$

In order to find a general equation for the time evolution of the second-order cross-moments $\langle s_i s_j \rangle$ [equation (5) in the main text] we proceed in a similar way as we did in the previous section for the first-order moments. Taking into account the master equation (S6) and using the definition of the average value in (S14), we can write for the second-order cross-moments,

$$\begin{aligned} \frac{d\langle s_i s_j \rangle}{dt} &= \sum_{\{s\}} s_i s_j \frac{dP(s_1,\dots,s_N)}{dt} \\ &= \sum_{\{s\}} \sum_{k=1}^{N} s_i s_j \left(E_k^{+1}-1\right)\left[s_k r_k^- P(s_1,\dots,s_N)\right] + \sum_{\{s\}} \sum_{k=1}^{N} s_i s_j \left(E_k^{-1}-1\right)\left[(1-s_k) r_k^+ P(s_1,\dots,s_N)\right] . \end{aligned} \tag{S21}$$

For the terms of the sum with $k \neq i, j$, we can use relation (S13) to write

$$\sum_{k\neq i,j}^{N} \sum_{\{s\}_k} s_i s_j \left[\left(\sum_{s_k} \left(E_k^{+1}-1\right)\left[s_k r_k^- P(s_1,\dots,s_N)\right] \right) + \left(\sum_{s_k} \left(E_k^{-1}-1\right)\left[(1-s_k) r_k^+ P(s_1,\dots,s_N)\right] \right) \right] = 0, \tag{S22}$$

where the equality follows from an application of relations (S7) and (S8). Similarly, we can use relations (S9) and (S10) to transform, in equation (S21), the terms with $k = i \neq j$ as

$$\begin{aligned} &\sum_{\{s\}_i} s_j \left[\left(\sum_{s_i} s_i \left(E_i^{+1}-1\right)\left[s_i r_i^- P(s_1,\dots,s_N)\right] \right) + \left(\sum_{s_i} s_i \left(E_i^{-1}-1\right)\left[(1-s_i) r_i^+ P(s_1,\dots,s_N)\right] \right) \right] \\ &= \sum_{\{s\}_i} s_j \left[-\sum_{s_i} s_i r_i^- P(s_1,\dots,s_N) + \sum_{s_i} (1-s_i) r_i^+ P(s_1,\dots,s_N) \right] \\ &= -\sum_{\{s\}} s_i s_j r_i^- P(s_1,\dots,s_N) + \sum_{\{s\}} (1-s_i) s_j r_i^+ P(s_1,\dots,s_N) \\ &= \langle r_i^+ s_j \rangle - \langle (r_i^+ + r_i^-) s_i s_j \rangle , \end{aligned} \tag{S23}$$

and, equivalently, the terms with $k = j \neq i$ as

$$\begin{aligned} &\sum_{\{s\}_j} s_i \left[\left(\sum_{s_j} s_j \left(E_j^{+1}-1\right)\left[s_j r_j^- P(s_1,\dots,s_N)\right] \right) + \left(\sum_{s_j} s_j \left(E_j^{-1}-1\right)\left[(1-s_j) r_j^+ P(s_1,\dots,s_N)\right] \right) \right] \\ &= \langle r_j^+ s_i \rangle - \langle (r_j^+ + r_j^-) s_i s_j \rangle . \end{aligned} \tag{S24}$$

Note that, for both expressions (S23) and (S24), we have assumed that $i \neq j$. In order to study the other case, when $i = j$, we simply need to notice that, being the possible values of the variables $s_i = \{0,1\}$, then $s_i^2 = s_i$, and therefore

$$\frac{d\langle s_i s_i \rangle}{dt} = \frac{d\langle s_i \rangle}{dt} = \langle r_i^+ \rangle - \langle (r_i^+ + r_i^-) s_i \rangle , \tag{S25}$$

where we have used the result (S20) for the first-order moments derived in the previous section.

Thus, we can write an equation for the second-order cross-moments as

$$\frac{d\langle s_i s_j \rangle}{dt} = \begin{cases} \langle r_i^+ s_j \rangle + \langle r_j^+ s_i \rangle - \langle q_{ij} s_i s_j \rangle & \text{if } i \neq j \\ \langle r_i^+ \rangle - \langle (r_i^+ + r_i^-) s_i \rangle & \text{if } i = j \end{cases} , \tag{S26}$$

where $q_{ij} = r_i^+ + r_i^- + r_j^+ + r_j^-$. Finally, using the Kronecker delta, we obtain the expression presented in the main text,

$$\frac{d\langle s_i s_j \rangle}{dt} = \langle r_i^+ s_j \rangle + \langle r_j^+ s_i \rangle - \langle q_{ij} s_i s_j \rangle + \delta_{ij} \left[\langle s_i r_i^- \rangle + \langle (1-s_i) r_i^+ \rangle \right] . \tag{S27}$$

4 Variance of n

We derive here an analytical expression for the steady state variance of n [equation (14) in the main text]. Let us start by introducing the transition rates of the noisy voter model [equation (1) in the main text] into the equation for the time evolution of the second-order cross-moments obtained in the previous section, equation (S27),

$$\begin{aligned}\frac{d\langle s_i s_j\rangle}{dt} =& a(\langle s_i\rangle + \langle s_j\rangle) + \frac{h}{k_i}\sum_{m\in nn(i)}\langle s_m s_j\rangle + \frac{h}{k_j}\sum_{m\in nn(j)}\langle s_m s_i\rangle - 2(2a+h)\langle s_i s_j\rangle \\ &+ \delta_{ij}\left[a + h\langle s_i\rangle + \frac{h}{k_i}\sum_{m\in nn(i)}\langle s_m\rangle - \frac{2h}{k_i}\sum_{m\in nn(i)}\langle s_m s_i\rangle\right].\end{aligned} \tag{S28}$$

Applying now the annealed approximation for uncorrelated networks described in the main text [see equation (10)], we can replace the sums over sets of neighbors by sums over the whole system, finding

$$\begin{aligned}\frac{d\langle s_i s_j\rangle}{dt} =& a(\langle s_i\rangle + \langle s_j\rangle) + \frac{h}{N\bar{k}}\sum_m k_m\left(\langle s_m s_i\rangle + \langle s_m s_j\rangle\right) - 2(2a+h)\langle s_i s_j\rangle \\ &+ \delta_{ij}\left[a + h\langle s_i\rangle + \frac{h}{N\bar{k}}\sum_m k_m\langle s_m\rangle - \frac{2h}{N\bar{k}}\sum_m k_m\langle s_m s_i\rangle\right].\end{aligned} \tag{S29}$$

Bearing in mind the definition of the covariance matrix, $\sigma_{ij} = \langle s_i s_j\rangle - \langle s_i\rangle\langle s_j\rangle$, we can find an equation for its time evolution from equation (6) in the main text and equation (S29),

$$\begin{aligned}\frac{d\sigma_{ij}}{dt} =& \frac{d\langle s_i s_j\rangle}{dt} - \frac{d\langle s_i\rangle}{dt}\langle s_j\rangle - \langle s_i\rangle\frac{d\langle s_j\rangle}{dt} \\ =& -2(2a+h)(\langle s_i s_j\rangle - \langle s_i\rangle\langle s_j\rangle) + \frac{h}{N\bar{k}}\sum_m k_m\left[(\langle s_m s_i\rangle - \langle s_m\rangle\langle s_i\rangle) + (\langle s_m s_j\rangle - \langle s_m\rangle\langle s_j\rangle)\right] \\ &+ \delta_{ij}\left[a + h\langle s_i\rangle + \frac{h}{N\bar{k}}\sum_m k_m\langle s_m\rangle - \frac{2h}{N\bar{k}}\sum_m k_m\langle s_m s_i\rangle\right],\end{aligned} \tag{S30}$$

which can be written in terms of only the covariance matrix and the first moments,

$$\begin{aligned}\frac{d\sigma_{ij}}{dt} =& -2(2a+h)\sigma_{ij} + \frac{h}{N\bar{k}}\sum_m k_m\left(\sigma_{mi} + \sigma_{mj}\right) \\ &+ \delta_{ij}\left[a + \frac{h}{N\bar{k}}\sum_m k_m\langle s_m\rangle + \left(h - \frac{2h}{N\bar{k}}\sum_m k_m\langle s_m\rangle\right)\langle s_i\rangle - \frac{2h}{N\bar{k}}\sum_m k_m\sigma_{mi}\right].\end{aligned} \tag{S31}$$

In the steady state, and using also the steady state solution of the first order moments $\langle s_i\rangle_{st} = 1/2$ [equation (7) in the main text], we find

$$\sigma_{ij} = \frac{\frac{h}{N\bar{k}}\sum_m k_m\left(\sigma_{mi} + \sigma_{mj}\right) + \delta_{ij}\left[a + \frac{h}{2} - \frac{2h}{N\bar{k}}\sum_m k_m\sigma_{mi}\right]}{2(2a+h)}. \tag{S32}$$

Note that, for the sake of notational simplicity, we have dropped the subindex st for the steady state solution of the covariance matrix. Recalling now the relation between the variance of n and the covariance matrix [equation (13) in the main text], we can

find an equation for the steady state variance of n by simply summing equation (S32) over i and j,

$$\begin{aligned}\sigma_{st}^2[n] = \sum_{ij}\sigma_{ij} &= \frac{\frac{h}{N\bar{k}}\sum_{ijm}k_m\left(\sigma_{mi}+\sigma_{mj}\right)+\sum_i\left[a+\frac{h}{2}-\frac{2h}{N\bar{k}}\sum_m k_m\sigma_{mi}\right]}{2(2a+h)}\\ &= \frac{\frac{h}{\bar{k}}\left(\sum_{im}k_m\sigma_{mi}+\sum_{jm}k_m\sigma_{mj}\right)+N\left(a+\frac{h}{2}\right)-\frac{2h}{N\bar{k}}\sum_{im}k_m\sigma_{mi}}{2(2a+h)}\\ &= \frac{N\left(a+\frac{h}{2}\right)+\frac{2h}{\bar{k}}\left(1-\frac{1}{N}\right)\sum_{im}k_m\sigma_{mi}}{2(2a+h)}\,.\end{aligned} \tag{S33}$$

Let us introduce now the set of variables S_x, with $x\in\{0,1,2,\ldots\}$, and defined as

$$S_x = \sum_{im}k_i^x k_m\sigma_{mi}\,. \tag{S34}$$

In this way, we can rewrite the steady state variance of n in terms of one of these new variables, S_0,

$$\sigma_{st}^2[n] = \frac{N\left(a+\frac{h}{2}\right)+\frac{2h}{\bar{k}}\left(1-\frac{1}{N}\right)S_0}{2(2a+h)}\,. \tag{S35}$$

In order to find an equation for this new variable S_0, we could use again the equation for the covariance matrix in (S32), multiplying it by k_j and summing over i and j, obtaining a solution in terms of the variable S_1. We could then proceed similarly and find an equation for S_1 as a function of S_2, for S_3 as a function of S_4, and so forth. In general, for any x, we have

$$\begin{aligned}S_x = \sum_{ij}k_i^x k_j\sigma_{ij} &= \frac{\frac{h}{N\bar{k}}\sum_{ijm}k_i^x k_j k_m\left(\sigma_{mi}+\sigma_{mj}\right)+\sum_i k_i^{x+1}\left[a+\frac{h}{2}-\frac{2h}{N\bar{k}}\sum_m k_m\sigma_{mi}\right]}{2(2a+h)}\\ &= \frac{\frac{h}{N\bar{k}}\sum_j k_j\sum_{im}k_i^x k_m\sigma_{mi}+\frac{h}{N\bar{k}}\sum_i k_i^x\sum_{jm}k_j k_m\sigma_{mj}+\sum_i k_i^{x+1}\left(a+\frac{h}{2}\right)-\frac{2h}{N\bar{k}}\sum_{im}k_i^{x+1}k_m\sigma_{mi}}{2(2a+h)}\\ &= \frac{hS_x+\frac{h}{\bar{k}}\overline{k^x}S_1+N\overline{k^{x+1}}\left(a+\frac{h}{2}\right)-\frac{2h}{N\bar{k}}S_{x+1}}{2(2a+h)}\,,\end{aligned} \tag{S36}$$

where the overbar notation is used for averages over the degree distribution [see equation (3) in the main text]. From equation (S36) we can obtain an expression for the variable S_x in terms of only S_1 and S_{x+1},

$$S_x = \frac{\frac{h}{\bar{k}}\overline{k^x}S_1+N\overline{k^{x+1}}\left(a+\frac{h}{2}\right)-\frac{2h}{N\bar{k}}S_{x+1}}{4a+h}\,. \tag{S37}$$

By inverting equation (S37), we can write all variables S_{x+1} in terms of the preceding ones,

$$S_{x+1} = \left[-\frac{(4a+h)N\bar{k}}{2h}\right]S_x+\frac{N}{2}\left[\overline{k^x}S_1+\frac{N\bar{k}}{h}\left(a+\frac{h}{2}\right)\overline{k^{x+1}}\right], \tag{S38}$$

which has the general form

$$S_{x+1} = AS_x+B_x\,. \tag{S39}$$

It is easy to see that this recurrence relation has the solution

$$S_{x+1} = A^x S_1 + \sum_{m=1}^{x} A^{x-m} B_x\,, \tag{S40}$$

where the choice of S_1 instead of S_0 in the first term allows us to write all the variables S_{x+1} in terms of only one of them, S_1. Note that this choice is required by the presence of a term with S_1 inside B_x. Thus, we can write the solution for our original recurrence relation in (S38) as

$$S_{x+1} = \left[-\frac{(4a+h)N\bar{k}}{2h}\right]^x S_1 + \sum_{m=1}^{x}\left[-\frac{(4a+h)N\bar{k}}{2h}\right]^{x-m}\frac{N}{2}\left[\overline{k^m}S_1 + \frac{N\bar{k}}{h}\left(a+\frac{h}{2}\right)\overline{k^{m+1}}\right]. \tag{S41}$$

If we now rewrite equation (S41) as

$$\frac{S_{x+1}}{\left[-\frac{(4a+h)N\bar{k}}{2h}\right]^x} = S_1 + \sum_{m=1}^{x}\left[-\frac{(4a+h)N\bar{k}}{2h}\right]^{-m}\frac{N}{2}\left[\overline{k^m}S_1 + \frac{N\bar{k}}{h}\left(a+\frac{h}{2}\right)\overline{k^{m+1}}\right], \tag{S42}$$

we find that the left hand side of this equation vanishes in the limit of $x\to\infty$,

$$\lim_{x\to\infty}\frac{S_{x+1}}{\left[-\frac{(4a+h)N\bar{k}}{2h}\right]^x} = \lim_{x\to\infty}\frac{\sum_{ij}k_i^{x+1}k_j\sigma_{ij}}{\left[-\frac{(4a+h)N\bar{k}}{2h}\right]^x} = \left[-\frac{(4a+h)N\bar{k}}{2h}\right]\lim_{x\to\infty}\sum_{ij}\left[-\frac{2hk_i}{(4a+h)N\bar{k}}\right]^{x+1}k_j\sigma_{ij} = 0\,, \tag{S43}$$

where we have used the definition of the variables S_x given in equation (S34). A necessary and sufficient condition for the last equality in equation (S43) to hold is that

$$\forall i : \left|-\frac{2hk_i}{(4a+h)N\bar{k}}\right| < 1 \implies \forall i : k_i < \frac{(4a+h)N\bar{k}}{2h}\,, \tag{S44}$$

which is generally true and always true for $h>0$ and $\bar{k}\geq 2$. Thus, in the $x\to\infty$ limit, we can equate the right hand side of equation (S42) to zero,

$$S_1 + \left(\sum_{m=1}^{\infty}\left[-\frac{(4a+h)N\bar{k}}{2h}\right]^{-m}\frac{N}{2}\overline{k^m}\right)S_1 + \left(\sum_{m=1}^{\infty}\left[-\frac{(4a+h)N\bar{k}}{2h}\right]^{-m}\frac{N^2\bar{k}}{2h}\left(a+\frac{h}{2}\right)\overline{k^{m+1}}\right) = 0\,, \tag{S45}$$

and find, in this way, a solution for S_1,

$$S_1 = \frac{-\frac{N^2\bar{k}}{2h}\left(a+\frac{h}{2}\right)\sum_{m=1}^{\infty}\left[\frac{-2h}{(4a+h)N\bar{k}}\right]^m\overline{k^{m+1}}}{1+\frac{N}{2}\sum_{m=1}^{\infty}\left[\frac{-2h}{(4a+h)N\bar{k}}\right]^m\overline{k^m}}\,. \tag{S46}$$

Regarding the sums in equation (S46), we can use the sum of the geometric series

$$\sum_{m=1}^{\infty}A^m\overline{k^{m+z}} = \overline{k^z\sum_{m=1}^{\infty}A^m k^m} = \overline{\frac{Ak^{z+1}}{1-Ak}}\,,\quad \text{if}\quad |Ak|<1\,, \tag{S47}$$

where the condition of convergence is exactly the same as presented before in equation (S44), and thus generally true and always true for $h>0$ and $\bar{k}\geq 2$. In this way, applying the result (S47) to equation (S46) we have

$$S_1 = \frac{N^2\bar{k}\left(a+\frac{h}{2}\right)\overline{\left(\frac{k^2}{1+\frac{2hk}{(4a+h)N\bar{k}}}\right)}}{(4a+h)N\bar{k} - hN\overline{\left(\frac{k}{1+\frac{2hk}{(4a+h)N\bar{k}}}\right)}} = \frac{N^2\bar{k}\left(a+\frac{h}{2}\right)(4a+h)\overline{\left(\frac{k^2}{(4a+h)N\bar{k}+2hk}\right)}}{4a+h-\frac{h}{\bar{k}}\overline{\left(\frac{(4a+h)N\bar{k}k}{(4a+h)N\bar{k}+2hk}\right)}}\,, \tag{S48}$$

where the denominator can be rewritten as

$$
\begin{aligned}
4a+h-\frac{h}{\bar{k}}\overline{\left(\frac{(4a+h)N\bar{k}k}{(4a+h)N\bar{k}+2hk}\right)} &= 4a+\frac{h}{\bar{k}}\overline{\frac{[(4a+h)N\bar{k}+2hk]\bar{k}-(4a+h)N\bar{k}k}{(4a+h)N\bar{k}+2hk}} \\
&= 4a+\frac{h}{\bar{k}}\overline{\frac{[(4a+h)N\bar{k}+2hk](\bar{k}-k)+2hk^2}{(4a+h)N\bar{k}+2hk}} \\
&= 4a+\frac{h}{\bar{k}}\overline{(\bar{k}-k)}+\frac{2h^2}{\bar{k}}\overline{\left(\frac{k^2}{(4a+h)N\bar{k}+2hk}\right)} \\
&= 4a+\frac{2h^2}{\bar{k}}\overline{\left(\frac{k^2}{(4a+h)N\bar{k}+2hk}\right)},
\end{aligned}
\tag{S49}
$$

thereby finding a final expression for S_1,

$$
S_1=\frac{N^2\bar{k}\left(a+\frac{h}{2}\right)(4a+h)\overline{\left(\frac{k^2}{(4a+h)N\bar{k}+2hk}\right)}}{4a+\frac{2h^2}{\bar{k}}\overline{\left(\frac{k^2}{(4a+h)N\bar{k}+2hk}\right)}}.
\tag{S50}
$$

If we now go back to the equation for the steady state variance $\sigma^2_{st}[n]$ as a function of S_0, equation (S35), and we use equation (S37) to find an expression for S_0 as a function of S_1,

$$
S_0=\frac{N\bar{k}\left(a+\frac{h}{2}\right)+\frac{h}{\bar{k}}\left(1-\frac{2}{N}\right)S_1}{4a+h},
\tag{S51}
$$

then we can write an equation for the steady state variance as a function of S_1,

$$
\sigma^2_{st}[n]=\frac{N}{4}\left[1+\frac{2h\left(1-\frac{1}{N}\right)}{4a+h}+\left(N-3+\frac{2}{N}\right)\left(\frac{h}{\bar{k}}\right)^2\frac{2S_1}{N^2\left(a+\frac{h}{2}\right)(4a+h)}\right].
\tag{S52}
$$

Finally, introducing here what we found for S_1 in equation (S50), we arrive to the final expression for the steady state variance of the global variable n as presented in the main text,

$$
\sigma^2_{st}[n]=\frac{N}{4}\left[1+\frac{2h\left(1-\frac{1}{N}\right)}{4a+h}+\left(N-3+\frac{2}{N}\right)\frac{\left(\frac{h^2}{\bar{k}}\right)\overline{\left(\frac{k^2}{(4a+h)N\bar{k}+2hk}\right)}}{2a+\left(\frac{h^2}{\bar{k}}\right)\overline{\left(\frac{k^2}{(4a+h)N\bar{k}+2hk}\right)}}\right].
\tag{S53}
$$

5 Asymptotic approximations for the variance of n

We develop here a first-order approximation for the steady state variance of n with respect to the system size N. Given the dependence of the result of this approximation on the relationship between the system size N and the noise parameter a, we are forced to consider two different asymptotic approximation regimes: one for small a [corresponding to equation (16) in the main text] and the other for large a [corresponding to equation (17) in the main text].

Let us start by noticing that the structural constraint imposed by the annealed approximation for uncorrelated networks on the degrees of the network, $k_i<\sqrt{N\bar{k}}$, allows us to write equation (S53) as

$$
\sigma^2_{st}[n]=\frac{N}{4}\left[1+\frac{2h\left(1-\frac{1}{N}\right)}{4a+h}+\left(N-3+\frac{2}{N}\right)\frac{\left(\frac{h^2}{\bar{k}}\right)\overline{\left(\frac{k^2}{(4a+h)N\bar{k}\left(1+\mathcal{O}\left(N^{-1/2}\right)\right)}\right)}}{2a+\left(\frac{h^2}{\bar{k}}\right)\overline{\left(\frac{k^2}{(4a+h)N\bar{k}\left(1+\mathcal{O}\left(N^{-1/2}\right)\right)}\right)}}\right].
\tag{S54}
$$

In this way, we notice that, depending on the order of the product aN, the approximation of the third term in equation (S54) will lead to different results. In particular, when the noise parameter a is of order $\mathcal{O}(N^{-1})$ or smaller, then the product aN is, at most, of order $\mathcal{O}(N^0)$, and we can continue with the approximation as

$$\begin{aligned}\sigma_{st}^2[n] &= \frac{N}{4}\left[1+\frac{2h\left(1-\frac{1}{N}\right)}{4a+h}+\left(N-3+\frac{2}{N}\right)\left(\frac{\left(\frac{h^2}{\bar{k}}\right)\left(\frac{\overline{k^2}}{(4a+h)N\bar{k}}\right)}{2a+\left(\frac{h^2}{\bar{k}}\right)\left(\frac{\overline{k^2}}{(4a+h)N\bar{k}}\right)}+\mathcal{O}(N^{-1/2})\right)\right] \\ &= \frac{N}{4}\left[1+2\left(1-\frac{1}{N}\right)+\left(N-3+\frac{2}{N}\right)\left(\frac{h\left(\frac{\overline{k^2}}{\bar{k}^2}\right)}{2aN+h\left(\frac{\overline{k^2}}{\bar{k}^2}\right)}+\mathcal{O}(N^{-1/2})\right)\right],\end{aligned} \tag{S55}$$

which, to the first order in N, becomes

$$\sigma_{st}^2[n] = \frac{N}{4}\left[N\left(\frac{h\left(\frac{\overline{k^2}}{\bar{k}^2}\right)}{2aN+h\left(\frac{\overline{k^2}}{\bar{k}^2}\right)}+\mathcal{O}(N^{-1/2})\right)\right]. \tag{S56}$$

Using now the definition of the variance of the degree distribution, $\sigma_k^2 = \overline{k^2} - \bar{k}^2$, we find the approximation presented in the main text for the steady state variance of n for small a and to the first order in N,

$$\sigma_{st}^2[n] = \frac{N^2}{4}\left[\frac{h\left(\frac{\sigma_k^2}{\bar{k}^2}+1\right)}{2aN+h\left(\frac{\sigma_k^2}{\bar{k}^2}+1\right)}\right]+\mathcal{O}(N^{3/2}). \tag{S57}$$

Note that the remaining terms are *at most* of order $\mathcal{O}(N^{3/2})$.

On the contrary, when a is of order $\mathcal{O}(N^0)$ or larger, then the product aN is, at least, of order $\mathcal{O}(N)$, and we can approximate equation (S54) as

$$\begin{aligned}\sigma_{st}^2[n] &= \frac{N}{4}\left[1+\frac{2h\left(1-\frac{1}{N}\right)}{4a+h}+\left(N-3+\frac{2}{N}\right)\left(\frac{\left(\frac{h^2}{\bar{k}}\right)\left(\frac{\overline{k^2}}{(4a+h)N\bar{k}}\right)}{2a+\left(\frac{h^2}{\bar{k}}\right)\left(\frac{\overline{k^2}}{(4a+h)N\bar{k}}\right)}+\mathcal{O}(N^{-3/2})\right)\right] \\ &= \frac{N}{4}\left[1+\frac{2h\left(1-\frac{1}{N}\right)}{4a+h}+\left(N-3+\frac{2}{N}\right)\left(\frac{h^2\left(\frac{\overline{k^2}}{\bar{k}^2}\right)}{2a(4a+h)N+h^2\left(\frac{\overline{k^2}}{\bar{k}^2}\right)}+\mathcal{O}(N^{-3/2})\right)\right] \\ &= \frac{N}{4}\left[1+\frac{2h\left(1-\frac{1}{N}\right)}{4a+h}+\left(N-3+\frac{2}{N}\right)\left(\frac{h^2\left(\frac{\overline{k^2}}{\bar{k}^2}\right)}{2a(4a+h)N}+\mathcal{O}(N^{-3/2})\right)\right].\end{aligned} \tag{S58}$$

Note that the remaining terms are now one order of N smaller than in the previous approximation [equation (S55)]. To the first

order in N we have

$$\sigma_{st}^2[n] = \frac{N}{4}\left[1 + \frac{2h}{4a+h} + \frac{h^2\left(\frac{\overline{k^2}}{\overline{k}^2}\right)}{2a(4a+h)} + \mathscr{O}(N^{-1/2})\right] = \frac{N}{4}\left[1 + \frac{4ah + h^2\left(\frac{\overline{k^2} - \overline{k}^2 + \overline{k}^2}{\overline{k}^2}\right)}{2a(4a+h)} + \mathscr{O}(N^{-1/2})\right], \tag{S59}$$

and, finally, we find the approximation presented in the main text for the steady state variance of n for large a and to the first order in N,

$$\sigma_{st}^2[n] = \frac{N}{4}\left[1 + \frac{h}{2a} + \frac{h^2\frac{\sigma_k^2}{\overline{k}^2}}{2a(4a+h)}\right] + \mathscr{O}(N^{1/2}), \tag{S60}$$

where the remaining terms are *at most* of order $\mathscr{O}(N^{1/2})$.

6 Critical point approximation

In this section, we derive an analytical approximation for the critical point of the bimodal-unimodal transition [equation (18) in the main text], which can be defined as the relationship between the model parameters a and h leading the steady state variance of n to take the value $\sigma_{st}^2[n] = N(N+2)/12$, corresponding to a uniform distribution between 0 and N. In particular, bearing in mind that the critical value a_c of a fully-connected system is of order $\mathscr{O}(N^{-1})$ and that the change due to the network structure appears to be of order $\mathscr{O}(N^0)$ (see Fig. 2 in the main text), then we can expect the value of the critical point to be still of order $\mathscr{O}(N^{-1})$, and we can therefore use the small a asymptotic approximation in equation (S57),

$$\sigma_{st}^2[n] = \frac{N^2}{4}\left[\frac{h\left(\frac{\sigma_k^2}{\overline{k}^2}+1\right)}{2a_cN + h\left(\frac{\sigma_k^2}{\overline{k}^2}+1\right)}\right] + \mathscr{O}(N^{3/2}) = \frac{N(N+2)}{12}. \tag{S61}$$

The solution of this equation leads to the, for large N, leads to the value of the critical point discussed in the main text,

$$a_c = \frac{h}{N}\left(\frac{\sigma_k^2}{\overline{k}^2}+1\right) + \mathscr{O}(N^{-3/2}), \tag{S62}$$

consistent with the assumption of a critical value of order $\mathscr{O}(N^{-1})$. Note that assuming, instead, the critical value to be of order $\mathscr{O}(N^0)$, and using therefore the large a asymptotic approximation in equation (S60), leads again to an a_c of order $\mathscr{O}(N^{-1})$, inconsistent with the initial assumption.

7 Order parameter: the interface density ρ

We obtain, this section, an analytical expression for the order parameter ρ [equation (21) in the main text]. ρ is defined as the interface density or density of active links, that is, the fraction of links connecting nodes in different states. In terms of the connectivity matrix A_{ij},

$$\rho = \frac{\frac{1}{2}\sum_{ij} A_{ij}[s_i(1-s_j) + (1-s_i)s_j]}{\frac{1}{2}\sum_{ij} A_{ij}} = \frac{\sum_{ij} A_{ij}(s_i + s_j - s_is_j)}{\sum_{ij} A_{ij}}, \tag{S63}$$

and introducing the annealed approximation for uncorrelated networks described in the main text [see equation (10) in the main text], we find

$$\rho = \frac{\sum_{ij} \frac{k_ik_j}{N\overline{k}}(s_i + s_j - s_is_j)}{\sum_{ij} \frac{k_ik_j}{N\overline{k}}} = \sum_{ij} \frac{k_ik_j}{(N\overline{k})^2}(s_i + s_j - s_is_j). \tag{S64}$$

Restricting our attention to the steady state average value of equation (S64),

$$\langle\rho\rangle_{st} = \sum_{ij} \frac{k_i k_j}{(N\bar{k})^2} \left(\langle s_i\rangle_{st} + \langle s_j\rangle_{st} - \langle s_i s_j\rangle_{st}\right), \tag{S65}$$

we can use the steady state mean solution found before for the individual node variables s_i, $\langle s_i\rangle_{st} = 1/2$, and the definition of the covariance matrix in the steady state, $\sigma_{ij} = \langle s_i s_j\rangle_{st} - 1/4$, in order to write

$$\langle\rho\rangle_{st} = \frac{1}{2} - \frac{2}{(N\bar{k})^2} \sum_{ij} k_i k_j \sigma_{ij}, \tag{S66}$$

where we can identify the variable S_1 [see equation (S34)],

$$\langle\rho\rangle_{st} = \frac{1}{2} - \frac{2S_1}{(N\bar{k})^2}. \tag{S67}$$

Finally, reversing the relation (S52) between the variance of n and the variable S_1, we can write the steady state average interface density ρ in terms of the variance of n,

$$\langle\rho\rangle_{st} = \frac{1}{2} - \frac{2}{(hN)^2} \left[\frac{(4a+h)(2a+h)}{\left(1-\frac{1}{N}\right)\left(1-\frac{2}{N}\right)} \left(\sigma^2[n] - \frac{N}{4}\right) - \frac{\left(a+\frac{h}{2}\right)}{\left(1-\frac{2}{N}\right)} hN \right], \tag{S68}$$

as it appears in the main text.

8 Autocorrelation function of n

We derive here an analytical expression for the steady state autocorrelation function of n [equations (23) and (24) in the main text], defined as

$$K_{st}[n](\tau) = \langle n(t+\tau)n(t)\rangle_{st} - \langle n\rangle_{st}^2, \tag{S69}$$

where τ plays the role of a time-lag. As far as the second point in time, $t+\tau$, is concerned, we assume that the system was at $n(t)$ at time t, and hence we can treat $n(t)$ as an initial condition,

$$K_{st}[n](\tau) = \langle\langle n(t+\tau)|n(t)\rangle n(t)\rangle_{st} - \langle n\rangle_{st}^2, \tag{S70}$$

which, in terms of the individual variables $\{s_i\}$ and taking into account that $\langle n\rangle_{st} = N/2$, can be written as

$$K_{st}[n](\tau) = \sum_{ij} \langle\langle s_i(t+\tau)|\{s_l(t)\}\rangle s_j(t)\rangle_{st} - \frac{N^2}{4}. \tag{S71}$$

We need, therefore, an expression for $\langle s_i(t+\tau)|\{s_l(t)\}\rangle$, which we find by integration of the equation for the temporal evolution of the first-order moments $\langle s_i\rangle$ —obtained by introducing the transition rates of the noisy voter model into equation (S20)—,

$$\frac{d\langle s_i(t+\tau)|\{s_l(t)\}\rangle}{d\tau} = a - (2a+h)\langle s_i(t+\tau)|\{s_l(t)\}\rangle + \frac{h}{N\bar{k}} \sum_m k_m \langle s_m(t+\tau)|\{s_l(t)\}\rangle. \tag{S72}$$

In order to integrate equation (S72), we must first obtain an expression for

$$b(t+\tau) \equiv \frac{h}{N\bar{k}} \sum_m k_m \langle s_m(t+\tau)|\{s_l(t)\}\rangle, \tag{S73}$$

which we can find by multiplying equation (S72) by $hk_i/N\bar{k}$ and summing over i,

$$\begin{aligned} \frac{d}{d\tau}\left(\frac{h}{N\bar{k}} \sum_i k_i \langle s_i(t+\tau)|\{s_l(t)\}\rangle\right) =& \frac{ah}{N\bar{k}} \sum_i k_i - \frac{(2a+h)h}{N\bar{k}} \sum_i k_i \langle s_i(t+\tau)|\{s_l(t)\}\rangle \\ &+ \left(\frac{h}{N\bar{k}}\right)^2 \sum_i k_i \sum_m k_m \langle s_m(t+\tau)|\{s_l(t)\}\rangle. \end{aligned} \tag{S74}$$

In this way, we arrive to the differential equation

$$\frac{db(t+\tau)}{d\tau} = ah - (2a+h)b(t+\tau) + hb(t+\tau) = ah - 2ab(t+\tau)\,, \tag{S75}$$

which has the solution

$$b(t+\tau) = \frac{h}{2}\left(1-e^{-2a\tau}\right) + b(t)e^{-2a\tau}\,, \tag{S76}$$

depending on the initial condition $b(t)$. Using this expression, we can now integrate equation (S72) for the first-order moments,

$$\frac{d\langle s_i(t+\tau)|\{s_l(t)\}\rangle}{d\tau} = a - (2a+h)\langle s_i(t+\tau)|\{s_l(t)\}\rangle + b(t+\tau)\,, \tag{S77}$$

which has the general solution

$$\begin{aligned}
\langle s_i(t+\tau)|\{s_l(t)\}\rangle &= \frac{\int_0^\tau e^{(2a+h)\tau'}\left[a+b(t+\tau')\right]d\tau' + c_1}{e^{(2a+h)\tau}} \\
&= \frac{\int_0^\tau e^{(2a+h)\tau'}\left[a+\frac{h}{2}\left(1-e^{-2a\tau'}\right)+b(t)e^{-2a\tau'}\right]d\tau' + c_1}{e^{(2a+h)\tau}} \\
&= \frac{\left(a+\frac{h}{2}\right)\int_0^\tau e^{(2a+h)\tau'}d\tau' + \left(b(t)-\frac{h}{2}\right)\int_0^\tau e^{h\tau'}d\tau' + c_1}{e^{(2a+h)\tau}} \\
&= \frac{1}{2}\left(1-e^{-(2a+h)\tau}\right) + \frac{b(t)-\frac{h}{2}}{h}\left(e^{-2a\tau}-e^{-(2a+h)\tau}\right) + c_1 e^{-(2a+h)\tau}\,.
\end{aligned} \tag{S78}$$

Applying now the initial condition $\langle s_i(t)|\{s_l(t)\}\rangle = s_i(t)$, we find

$$\langle s_i(t+\tau)|\{s_l(t)\}\rangle = \frac{1}{2}\left(1-e^{-(2a+h)\tau}\right) + \frac{b(t)-\frac{h}{2}}{h}\left(e^{-2a\tau}-e^{-(2a+h)\tau}\right) + s_i(t)e^{-(2a+h)\tau}\,. \tag{S79}$$

We are now ready to go back to the autocorrelation function (S71) and write, in the steady state,

$$\begin{aligned}
K_{st}[n](\tau) =& \sum_{ij}\left\langle \frac{1}{2}\left(1-e^{-(2a+h)\tau}\right)s_j(t)\right\rangle_{st} + \sum_{ij}\left\langle \frac{b(t)-\frac{h}{2}}{h}\left(e^{-2a\tau}-e^{-(2a+h)\tau}\right)s_j(t)\right\rangle_{st} \\
&+ \sum_{ij}\left\langle s_i(t)s_j(t)e^{-(2a+h)\tau}\right\rangle_{st} - \frac{N^2}{4}\,.
\end{aligned} \tag{S80}$$

Given that we assume the state of the system at t to be our initial condition, $b(t)$ can be written as

$$b(t) = \frac{h}{N\bar{k}}\sum_i k_i\langle s_i(t)|\{s_l(t)\}\rangle = \frac{h}{N\bar{k}}\sum_i k_i s_i(t)\,, \tag{S81}$$

and thus we have, for the autocorrelation function,

$$\begin{aligned}
K_{st}[n](\tau) =& \frac{1}{2}\left(1-e^{-(2a+h)\tau}\right)\sum_{ij}\langle s_j(t)\rangle_{st} + \frac{1}{N\bar{k}}\left(e^{-2a\tau}-e^{-(2a+h)\tau}\right)\sum_{ijm}k_m\langle s_m(t)s_j(t)\rangle_{st} \\
&- \frac{1}{2}\left(e^{-2a\tau}-e^{-(2a+h)\tau}\right)\sum_{ij}\langle s_j(t)\rangle_{st} + e^{-(2a+h)\tau}\sum_{ij}\langle s_i(t)s_j(t)\rangle_{st} - \frac{N^2}{4}\,.
\end{aligned} \tag{S82}$$

Using now the value found before for the steady state solution of the first-order moments, $\langle s_i\rangle_{st} = 1/2$, and the definition of the covariance matrix in the steady state, $\sigma_{ij} = \langle s_i s_j\rangle_{st} - \langle s_i\rangle_{st}^2 = \langle s_i s_j\rangle_{st} - 1/4$, we find

$$K_{st}[n](\tau) = -e^{-2a\tau}\frac{N^2}{4} + \frac{1}{\bar{k}}\left(e^{-2a\tau}-e^{-(2a+h)\tau}\right)\sum_{jm}k_m\left(\sigma_{mj}+\frac{1}{4}\right) + e^{-(2a+h)\tau}\sum_{ij}\left(\sigma_{ij}+\frac{1}{4}\right)\,. \tag{S83}$$

Finally, identifying in the previous equation the variance of n and the variable S_1 [see equation (S34)], and reordering terms according to their exponential decay, we find the expression for the autocorrelation function of n discussed in the main text,

$$K_{st}[n](\tau) = \left(\sigma^2[n] - \frac{S_1}{\overline{k}}\right) e^{-(2a+h)\tau} + \frac{S_1}{\overline{k}} e^{-2a\tau}. \qquad \text{(S84)}$$

The definition of the variable S_1 given in the main text, as a function of the variance of n, can be directly obtained by reversing equation (S52).

9 Suppementary Figure S1

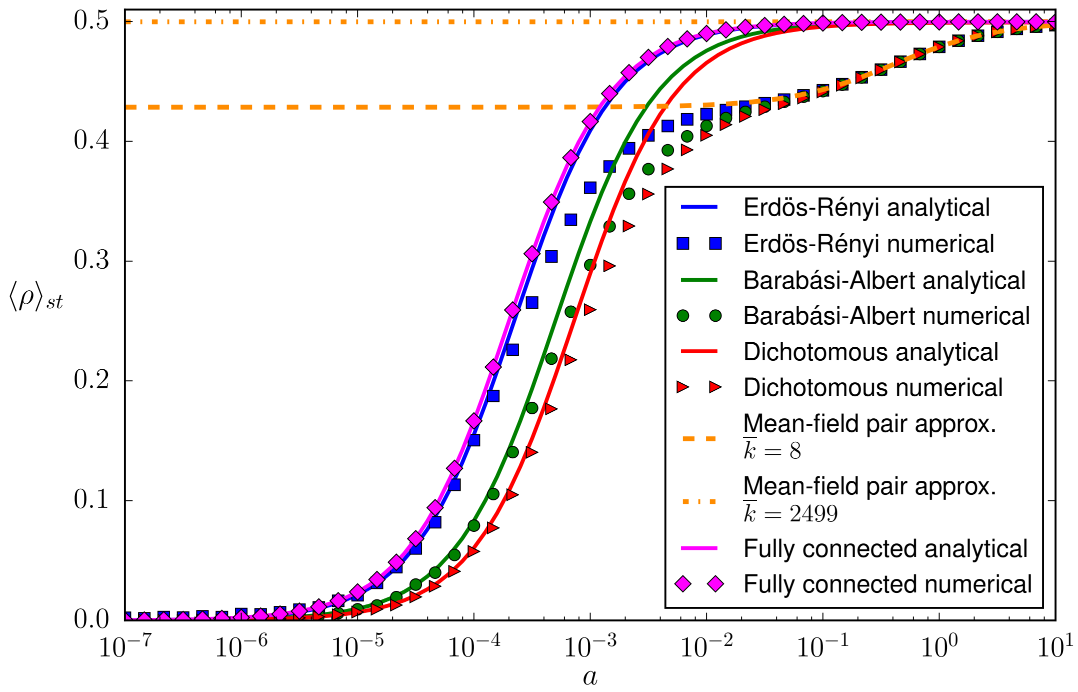

Figure S1. Steady state of the average interface density as a function of the noise parameter a in a linear-logarithmic scale and for three different types of networks with mean degree $\overline{k} = 8$: Erdös-Rényi random network, Barabási-Albert scale-free network and dichotomous network. A fully connected topology is also included for comparisson. Symbols: Numerical results (averages over 20 networks, 10 realizations per network and 50000 time steps per realization). Solid lines: Analytical results [see equation (S68)]. Dashed line: Mean-field pair-approximation (see[1]) for a mean degree $\overline{k} = 8$. Dash-dotted line: Mean-field pair-approximation for a mean degree $\overline{k} = 2499$. The interaction parameter is fixed as $h = 1$ and the system size as $N = 2500$.

References

1. Diakonova, M., Eguíluz, V. M. & San Miguel, M. Noise in coevolving networks. *Phys. Rev. E* **92**, 032803 (2015).
